# Burnout and patient safety: A discriminant analysis of paediatric nurses by low to high managerial support

**DOI:** 10.1002/nop2.708

**Published:** 2020-12-04

**Authors:** Haitham Khatatbeh, Annamária Pakai, Dorina Pusztai, Szilvia Szunomár, Noémi Fullér, Gyula Kovács Szebeni, Adrienn Siket, Miklós Zrínyi, András Oláh

**Affiliations:** ^1^ Faculty of Health Sciences University of Pécs Pécs Hungary; ^2^ Faculty of Health Sciences University of Debrecen Nyiregyhaza Hungary

**Keywords:** adverse events, burnout, nurse manager, quality of life, manager support

## Abstract

**Aim:**

To explore how levels of managerial support discriminate paediatric nurses' burnout, quality of life, intent to leave and adverse patient events.

**Design:**

A quantitative correlational study.

**Methods:**

A total of 225 paediatric nurses were selected from nine major hospitals across Jordan. The main measures used were the Copenhagen Burnout Inventory and the brief version of World Health Organization‐Quality of Life Instrument. The study methods were compliant with the STROBE checklist.

**Results:**

Nurse manager support was negatively associated with adverse patient events, work‐related burnout, client‐related burnout, and intent to leave; and positively with physical and psychological quality of life. Frequency of nosocomial infections characterized low manager support, whereas medication errors described high support. Greater nurse manager support decreased the likelihood of adverse patient outcomes.

## 1 INTRODUCTION

In essence, nurse manager support is one of the most critical aspects of today's professional commitments and has multiple functions. By being able to buffer work‐related stress, restore work–life balance and manage burnout, nurse managers preserve the quality and safety of paediatric care. On the individual level, nurse managers contribute to a healthier workforce by decreasing psychosomatic symptoms and diseases that are the direct consequence of high‐intensity environments and heavy workloads. Finally, nurse managers uniquely lower staff fluctuation and sustain a healthy workforce by improving job satisfaction and work‐related quality of life. However, we have limited research concerning the characteristics and influence of low and high managerial support perceived by paediatric nurses.

## 2 BACKGROUND

Health systems are challenged globally to constantly adapt to the rising number of patients, and nurse managers have been defined as “change agents” or “change coaches” of that adaptation process (Stefancyk et al., 2013). Among others, a key competency of a change agent is being able to instil hope and provide solid and continuous support to colleagues. Why support is so critical has been argued by Kramer and colleagues (2007), who stated that staff support is the cornerstone of any productive and healthy work environment. That is, nurse managers who wish to attain task accomplishment as a primary goal of their unit or organization will eventually not achieve optimal functioning of their staff (Cummings et al., 2010).

One aspect of that optimal functioning, where nurse manager support becomes critical, is ensuring patient safety. Merrill (2015) argued that transformational leadership and managerial support were strong foundations of a safety climate in nursing departments. More importantly, nurse manager ability and leadership support of frontline nurses were significant determinants of missed nursing care (Kim et al., 2018). Boamah et al., (2018) documented that transformational leadership and managerial support, through increased job satisfaction, were responsible for less frequent adverse patient incidents. However, the deficiency in nurse manager support can be a source of work‐related burnout, which has the potential to compromise quality patient outcomes (Nantsupawat et al., 2016). Researchers documented a unit increase of emotional burnout in their study to be associated with 2.63 odds of poor quality of care, a 47% rise in medication errors and a 32% increase in infection rates. Nurse managers, therefore, need to be coaches of their staff's well‐being to sustain a stable record of safe patient care.

Adams et al., (2019) claimed that unit managers' behaviour had a direct impact on their staff's well‐being and that managers have a fundamental role in supporting the quality of life of nurses. Quality of life has also been viewed as a major contributor to nurse retention (Adams et al., 2019; Nowrouzi et al., 2016). Work–life imbalance, a concept underlying both burnout and quality of life, was seen as a strong predictor of frequent thoughts of leaving the nursing profession (Hämmig, 2018). Spence Laschinger and colleagues (2012) also showed that increased perceptions of managerial support translated into improved quality of care and reduced nurses' intent to leave. At the same time, nurse manager behaviours that showed support for nurses in terms of personal communication, respect and creating a feeling of being cared for have resulted in higher levels of job satisfaction (Feather et al., 2015). Positive perceptions of work‐related quality of life (including managerial support) were powerful predictors of intent to stay on the job (Lee et al., 2015).

When nurse managers, using personal encouragement and professional support, were able to mitigate stress emanating from work–life imbalances, they considerably increased staff resilience and retention (Kim & Windsor, 2015). Creating healthy work environments (reducing occupational stress and burnout) was seen as a way of improving nurses' work‐related quality of life, which in turn helps maintain safe patient care and arguably improves staff retention (Nowrouzi et al., 2015). Considering paediatric nurses specifically, the level of burnout was reported high, in the range of 21%–39% (Pradas‐Hernández et al., 2018). Knupp et al., (2018) linked lower exhaustion of neonatal nurses to greater manager support and better leadership skills. As for patient‐related adverse events, paediatric nurses were found to have a greater number of missed care activities under unsupportive working conditions (Lake et al., 2017). Better work environments for paediatric nurses reduced adverse patient outcomes by 40% (Lake et al., 2017). We saw that moral distress, often caused by inadequate managerial guidance and aid, was a strong determinant of paediatric nurse job exit (Dyo et al., 2016; Sannino et al., 2019). However, showing charismatic leadership was dissociated with the intent to leave paediatric nursing (Blake et al., 2013). Authors argue that for nurse managers to properly intervene, they have to know what factors staff nurses associate with low and high managerial support.

The aim of the current study, therefore, was to explore how levels of managerial support discriminate paediatric nurses on dimensions of burnout, quality of life, intent to leave and adverse patient events. The following research questions were investigated in this paper: are there differences on burnout, quality of life, intent to leave and adverse patient events by level of managerial support?, do levels of managerial support for paediatric nurses differ on burnout, quality of life, intent to leave and adverse patient events? and what factors will discriminate low, medium and high levels of managerial support?

## 3 METHODS

The research used a cross‐sectional, correlational/discriminatory approach. Respondents were approached on the day of work and asked to consent to participation. A total of nine hospitals were selected as research sites: eight Ministry of Health affiliated (one paediatric hospital); and a university hospital. Hospitals were nationally representative of high‐level care institutions since most people live in the north and central region of Jordan. Therefore, five hospitals were selected from the northern region, two hospitals from the central region and a hospital from the southern region. Hospitals in the private sector and military hospitals were excluded from this study.

Printed surveys were handed over to nurse managers, who distributed them to staff. Surveys were voluntary and anonymous; completed items were placed in a sealed envelope and returned to nurse managers. Surveys were collected by the researcher a day later. Staff was asked to fill surveys independently and not to discuss responses. Despite the anonymity, all participants were asked to sign a written consent. The necessary ethical approvals were obtained before research implementation. Data were collected between December 2019–March 2020. As instruments were available in the public domain, no prior permission for use was sought. Because English is the official language of nursing education in Jordan, the original English versions of the instruments were used. A pilot test was run on an initial sample of 35 nurses to assess ease of instrument implementation and any validity concern. The study methods were compliant with the STrengthening the Reporting of OBservational studies in Epidemiology (STROBE) checklist (Appendix S1).

### 3.1 Sample

An initial pool of 500 paediatric nurses were listed as potential participants. Out of this sample, 300 nurses had randomly been selected, and those meeting inclusion criteria and consenting to participation were approached by the research team via nurse managers in various units. Final participants, however, reflected a more convenient sampling outcome as nurses on duty on the day of data collection were ultimately involved. Inclusion criteria were (a) having worked for at least 1 year in a clinical paediatric nurse position prior to study; (b) holding at least an undergraduate nursing degree (vocational) as a minimum; (c) being on a permanent or annual job contract (part of the regular staff); (d) being employed by a university or ministry of health affiliated hospital; and (e) being a Jordanian citizen.

To ensure adequate statistical power, a priori sample size calculation was done (G*Power, 2020). Using a global MANOVA approach for discriminant analysis with medium effect size, significance set at 5%, power at 0.82, number of groups being three (dependent variables), and number of response variables being eight (independent variables), a total sample size of 168 patients was required. In the post hoc analysis, our final 225 patients provided a power of 0.93, sufficient to infer meaningful statistical conclusions.

### 3.2 Main instruments

To measure burnout, the Copenhagen Burnout Inventory (CBI) was used (Borritz et al., 2006). The CBI measures three dimensions of burnout: personal, work‐related and client‐related. The CBI measures on a Likert scale approach (never to always and very low degree to very high degree). Each subscale is scored individually, and the total score is the sum of final subscale scores. Higher scores indicate more burnout. Validity and reliability have been reported by Sestili et al. (2018), who found Cronbach's alpha 0.892, 0.868 and 0.836 for the three subscales, respectively. We obtained reliability of 0.905, 0.830 and 0.884 for the subscales in our research.

To evaluate quality of life, the brief version of the World Health Organization‐Quality of Life Instrument (WHOQOL‐BREF) was used (World Health Organization, 1996). It assesses the individual's perceptions in the context of their culture and value systems and their personal goals, standards and concerns. The WHOQOL‐BREF instrument comprises 26 items measuring four domains: physical health, psychological health, social relationships and environment. Items are measured on a 5‐point Likert scale (not at all, completely). Scores can be transformed to a 0–100 range scale; higher scores indicate better quality of life. The instrument has established validity, and reliability assessed as Cronbach's alpha was 0.921 in this research.

To assess adverse patient events, items from Cho et al., (2016) were adopted. Frequency of wrong medication dispensed, patient falls after admission, nosocomial infections and pressure ulcers were assessed on a 6‐point Likert scale (never, every day). Cho et al., (2016) refer to established validity and reliability in prior investigations; reliability for this study was 0.833.

Finally, job satisfaction was measured by asking patients how satisfied they had been with their current position (5‐point scale, very dissatisfied, very satisfied). Nurse manager support was assessed by asking the amount of personal and professional support received (5‐point scale, very weak, very strong). Time available for family and monthly salary perception were assessed as dichotomous variables (enough/not enough and meeting personal needs/not meeting personal needs). The perceived workload was measured on a 5‐point scale (very low, very high). Intent to leave nursing was assessed by asking respondents if they had considered leaving their current nursing job in the past month (no/yes). Self‐esteem was measured by asking patients how they rated their self‐worth (Compared to others I am a person of worth and have a positive attitude towards myself, 4‐point Likert scale, strongly disagree‐strongly agree).

### 3.3 Statistical analyses

Frequencies and descriptive analyses were done to describe date distributions. One sample Kolmogorov–Smirnov test was run to evaluate the normal distribution. Spearman correlations were employed to establish associations across variables. Discriminant analysis was done to evaluate characteristics of low versus high managerial support as it related to staff nurses. The level of significance was set at 5%; one‐tailed tests were used where applicable. SPSS version 25.0 for Mac was used to run the analyses. No data replacement policy was employed; items with missing data were excluded from analyses.

## 4 RESULTS

A total of 225 patients completed the survey (response rate of 75%). Most (95.1%) of the final sample was female and married (82.7%), a smaller proportion lived single (15.1%) or divorced/widowed (1.8% and 0.4%). Most respondents were employed on either permanent (78.5%) or annual (15.7%) job contracts. Level of education was tilted towards the bachelor's degree (87.6%), vocational training was 2.2%, while master's degree holders represented 10.2% of the sample. Nurses had 11.1 years (*SD* 6.74) of professional experience on average and had an average of 7.17 (*SD* 5.02) patients assigned to each of them. They slept an average of 6.51 (*SD* 1.66) hours daily. Table 1. displays descriptive statistics of main measures of interest.

**TABLE 1:** Descriptive data

	*N*	Minimum	Maximum	Mean	Std. deviation
Personal burnout (CBI)	224	16.67	100.00	73.77	20.88
Work‐related burnout (CBI)	225	17.86	100.00	66.05	18.60
Client‐related burnout (CBI)	225	0.00	100.00	62.50	21.54
Physical domain (WHOQOL‐BREF)	222	6.00	81.00	42.97	14.03
Psychological domain (WHOQOL‐BREF)	221	6.00	88.00	47.71	16.14
Social domain (WHOQOL‐BREF)	223	0.00	94.00	45.14	20.64
Environmental domain (WHOQOL‐BREF)	221	0.00	81.00	44.38	16.00
Perceived workload	222	1	5	4.07	0.89
Job satisfaction	225	1	23	3.02	1.72
Manager support	225	1	5	3.42	1.06
Perceived self‐esteem	225	1	4	3.14	0.79

As for thoughts about leaving the nursing profession, 48.2% reported having considered it during the last month (51.8% said they did not). Most respondents did not find monthly salary meeting their personal needs (72.2% versus 27.8% finding it enough to make a living). Considering time available for family, 81.8% declared “not enough time available” whereas 18.2% found it sufficient to satisfy family needs.

Since one sample K‐S tests confirmed non‐normal data distributions, Spearman correlation coefficients were used to determine associations between main measures. Table 2 presents correlations among managerial support and adverse patient events as perceived by paediatric nurses. Greater managerial support was significantly negatively associated with all indicators of adverse patient events; that is, greater support reduced the incidence of adverse patient events. Table 3 shows the correlations of managerial support across burnout and quality of life domains. Greater support resulted in a better quality of life, manager support showing the strongest positive association with physical health/quality of life.

**TABLE 2:** Correlations between managerial support and adverse patient events

	(1)	(2)	(3)	(4)	(5)
(1) Manager support	1.000	−.224**	−.249**	−.225**	−.148*
(2) How often patients receive wrong medication or dose during last month?		1.000	.665**	.627**	.534**
(3) How often patients experience pressure ulcer during last month?			1.000	.517**	.502**
(4) How often patients experience falls after admission during last month?				1.000	.449**
(5) How often patients experience nosocomial infection during last month?					1.000

* Correlation is significant at the 0.05 level (1‐tailed).

** Correlation is significant at the 0.01 level (1‐tailed).

**TABLE 3:** Correlations between managerial support,CBI subscales, and WHOQOL‐BREF domains

	Manager support	Personal burnout	Work‐related burnout	Client‐related burnout	Physical QOL	Psychological QOL	Social QOL	Environmental QOL
Manager support	1.000	−.140*	−.218**	−.224**	.251**	.238**	.231**	.207**
Personal burnout		1.000	.606**	.545**	−.475**	−.469**	−.371**	−.391**
Work‐related burnout			1.000	.676**	−.556**	−.453**	−.266**	−.497**
Client‐related burnout				1.000	−.514**	−.534**	−.354**	−.525**
Physical QOL					1.000	.571**	.483**	.600**
Psychological QOL						1.000	.617**	.747**
Social QOL							1.000	.655**
Environmental QOL								1.000

* Correlation is significant at the 0.05 level (1‐tailed).

** Correlation is significant at the 0.01 level (1‐tailed).

As for measures of burnout, managerial support was negatively associated with burnout; that is, greater support resulted in lower burnout. Most importantly, greater support showed the strongest inverse relationship to client‐related burnout (more support, less client‐related burnout) and the strongest positive to the physical quality of life (increased support, a better quality of life). Note that of all correlations, work‐related burnout had the greatest inverse association with the physical quality of life, that is, the greater work‐related burnout nurses experienced, the lower their physical health had been.

While not the main focus of this research, we found additional associations among other variables of interest. Managerial support was positively related to job satisfaction (*r* = .325, *p* < .001) and perceived self‐esteem (*r* = .205, *p* = .001) and negatively to exposure to reported patient violence (−0.149, *p* = .013). That is, increased managerial support boosted nurses' self‐esteem and enhanced satisfaction with their nursing position, and reduced the prevalence of violence towards nursing staff. Finally, intent to leave and perceived workload were both negatively correlated with managerial support. That is, greater managerial support decreased the probability that one would leave the nursing profession (*r* = −.151, *p* = .012). Greater support had a positive impact on perceived workload as well; workload was considered lower when higher support had been experienced.

To differentiate staff nurses' perceptions of low, medium and high managerial support, discriminant analysis was performed. Cut‐off scores from data were established to establish three groups of managerial support and then used as the dependent variable of interest. Table 4 shows the outcomes of the analysis. Of the two functions tested, first discriminant function was significant (Wilk's lambda = 0.82, *p* = .003). That is, 67% of the total variance in discriminant scores was accounted for by group differences in the predictor variables. The canonical correlation coefficient was 0.397; predictors accounted for 15.8% of the variance in group membership.

**TABLE 4:** Functions at group centroids and structure matrix

Structure matrix	Function	
	1	2
Physical QOL	.671*	0.178
Psychological QOL	.644*	−0.104
Work‐related burnout	−.635*	−0.305
Client‐related burnout	−.535*	0.025
Do you have thoughts of leaving nursing job	−.442*	0.175
How often patients experienced nosocomial infection during last month?	−.228*	−0.155
Time available for family	−0.034	.514*
Monthly salary perception	0.395	.471*
How often patients received wrong medication or dose during last month?	−0.141	.356*
Perceived workload	−0.018	−.341*

Group centroids	Function	
Manager support	1	2
Low	−0.825	−0.065
Medium	0.179	0.094
High	0.445	−0.458

* Largest absolute correlation between each variable and any discriminant function.

Examining the largest absolute value of group centroids in Table 4 informs about the characteristics of each discriminant function. Function 1 discriminates those with low manager support from medium and high support. Function 2, however, discriminates those with high support from the other two categories. The structure matrix shows the structure coefficients with their relative contribution to each discriminant function. Thus, function 1 (low support) is dominantly characterized by the physical and psychological quality of life and work‐related burnout. Client‐related burnout and intent to leave formed the second set of variables by magnitude. While being last, nosocomial infections were also associated with low managerial support. When squared, structure coefficients indicate the proportion of variance in the variable that is accounted for by the discriminant function. Thus, 45% of the variance in physical quality of life and 19.5% in intent to leave nursing were accounted for by the first function. The second function (high support) was characterized by time for family and financial compensation. While time available for the family was the dominant variable (26.4% in family time was account for by this function), medication errors were also characteristic of high managerial support.

## 5 DISCUSSION

The primary goal of the current research was to assess how low to high managerial support is associated with staff nurses' perceptions of burnout, quality of life, intent to leave and adverse patient events. The results of the discriminant analysis showed a clear difference between low and high managerial support. The low managerial support was dominantly associated with physical and psychological quality of life and work‐related burnout. High support, however, was characterized by the time available for family and, to a lesser extent, by financial compensation. Importantly enough, while intent to leave nursing was also associated with low managerial support, its relative magnitude made it less influential. Low support was responsible only in 19.5% for the variance in intent to leave. That is, low managerial support had a much stronger impact on the physical quality of life (45% shared variance) and work‐related burnout (28.6% shared variance). Examining the variables that were loaded under the low managerial support, we can organize variables into three major groups in the order of importance: (a) physical and psychological impact (burnout), (b) work‐related effects; and (c) adverse patient events. Note that items loaded under the low managerial support were highly correlated in the correlation matrix; therefore, making progress in improving the first set of variables should have a strong positive impact on the rest. While most managers would think that reducing the psychological toll should have the greatest influence on burnout, we showed that low managerial support was associated with psychical quality of life the strongest. This is an important finding in that when nurses perceive managerial support to be low, they associate this with a drop in physical quality of life in the first place; the psychological burden comes only in second. Therefore, one implication of the research was that nurse managers should focus on improving the physical quality of life, not the psychological burden of their staff because it had the strongest negative correlation with work‐related burnout. Also note that greater managerial support was linked to less client‐related burnout of staff, which is an important finding for managers to consider when aiming to improve the quality of paediatric care.

Time available for family dominantly characterized high managerial support. We found that greater managerial support was associated with “enough time for family.” High support was responsible for 26.4% of the variance in time for family. Since more time for family was associated with lower intent to leave nursing, the second implication of our findings was that nurse managers should focus increased attention on introducing even more flexibility in the organization of work for their staff. High managerial support was not perceived by staff as emotional or professional help but more as having sufficient time to spend with their families.

Finally, while we assumed the managerial support should only have an indirect influence on adverse patient events, our research, however, implied that greater managerial support had a direct negative impact on all dimensions of adverse patient events. While earlier reports linked quality of nursing care and staff–patient ratios and doctor–nurse relationships and nurse workload to adverse patient events (Cho et al., 2016; Kang et al., 2014, 2016; Lucero et al., 2010), our research uniquely established a direct relationship between nurse manager support and patient outcomes. Discriminant analysis results, however, separated adverse patient events between low and high support functions. Low managerial support was associated with more nosocomial infections, whereas high support with lower medication errors. That is, low support increased the frequency of nosocomial infections, whereas high support decreased medication errors. Since these findings are novel in nature, we suggest exploring the direct impact of managerial support on adverse patient events outside the paediatric nurse cohort in future research.

### 5.1 Limitations

The authors recognize that the final sample was rather conveniently selected. The authors also acknowledge that the study was restricted to paediatric nurses in Jordan, which may have a specific nurse manager culture not observed elsewhere. These issues may limit the immediate generalizability of results; replication is recommended in other healthcare contexts.

## 6 CONCLUSIONS AND IMPLICATIONS

Data confirmed that nurse manager support was key in decreasing adverse patient events. Discriminant analysis results showed high manager support associated with lower medication errors, whereas low manager support was linked to greater nosocomial infections. This implies that to further improve nosocomial infections and medication errors, unit nurse managers, besides re‐training of staff, need to strengthen the amount of support they provide to staff as it may be a critical, but potentially missed, link to achieve a better quality of care.

Our research has also demonstrated that low managerial support showed the strongest association with the physical quality of life. When we looked at the order of variables loaded under “low managerial support,” we concluded that managerial interventions should address improving the physical and psychological aspects of paediatric nurses' work‐related quality of life in the first place. Since the time available for family emerged as the dominant characteristic of high managerial support, authors suggest a joint intervention that considers results of both discriminant functions: improving physical and psychological quality of nurses work–life combined with enhanced flexibility of work to allow for more time spent with family (reduced workload, balanced shifts, limited nurse–patient ratios) should be perceived by staff nurses as signs of high managerial support, which in turn should significantly reduce staff fluctuations and increase patient safety.

## CONFLICT OF INTEREST

Authors report no conflict of interest related to either external or internal funding or any other sources that may have influenced research implementation or interpretation of results.

## AUTHOR CONTRIBUTIONS

All authors are responsible for the reported research and have approved the manuscript as submitted. Haitham Khatatbeh: Conceptualization, Data collection, Data curation, Writing—Original draft preparation; Annamária Pakai: Conceptualization, Methodology, Software, Supervision; Dorina Pusztai, Szilvia Szunomár, Noémi Fullér, Gyula Kovács Szebeni and Adrienn Siket: Conceptualization, Writing—Reviewing and Editing; Miklós Zrínyi: Methodology, Software, Writing—Original draft preparation, Reviewing and Editing; András Oláh: Supervision, Writing—Reviewing and Editing.

## ETHICAL APPROVAL

Ethical approval was obtained before research implementation both from the Scientific Research Committee of the Jordanian Ministry of Health (reg. # 21,114) and from the Ethics Committee of King Abdullah University Hospital (reg. # 13–3–17).

## Supporting information

App S1

## Data Availability

The raw data that support the results of this research are available from the corresponding author upon a reasonable request.
